# Lower versus higher hemoglobin threshold for transfusion in ARDS patients with and without ECMO

**DOI:** 10.1186/s13054-020-03405-4

**Published:** 2020-12-16

**Authors:** O. Hunsicker, L. Materne, V. Bünger, A. Krannich, F. Balzer, C. Spies, R. C. Francis, S. Weber-Carstens, M. Menk, J. A. Graw

**Affiliations:** 1 Department of Anesthesiology and Operative Intensive Care Medicine CCM / CVK Charité – Universitätsmedizin Berlin, corporate member of Freie Universität Berlin, Humboldt-Universität zu Berlin, and Berlin Institute of Health, Augustenburger Platz 1, 13353 Berlin, Germany; 2 ARDS/ECMO Centrum Charité, Charité - Universitätsmedizin Berlin, Berlin, Germany; 3 Clinical Trial Office, Charité - Universitätsmedizin Berlin, Berlin, Germany; 4 Berlin Institute of Health (BIH), Berlin, Germany

**Keywords:** Transfusion, Red blood cells, ARDS

## Abstract

**Background:**

Efficacy and safety of different hemoglobin thresholds for transfusion of red blood cells (RBCs) in adults with an acute respiratory distress syndrome (ARDS) are unknown. We therefore assessed the effect of two transfusion thresholds on short-term outcome in patients with ARDS.

**Methods:**

Patients who received transfusions of RBCs were identified from a cohort of 1044 ARDS patients. After propensity score matching, patients transfused at a hemoglobin concentration of 8 g/dl or less (lower-threshold) were compared to patients transfused at a hemoglobin concentration of 10 g/dl or less (higher-threshold). The primary endpoint was 28-day mortality. Secondary endpoints included ECMO-free, ventilator-free, sedation-free, and organ dysfunction-free composites.

**Measurements and main results:**

One hundred ninety-two patients were eligible for analysis of the matched cohort. Patients in the lower-threshold group had similar baseline characteristics and hemoglobin levels at ARDS onset but received fewer RBC units and had lower hemoglobin levels compared with the higher-threshold group during the course on the ICU (9.1 [IQR, 8.7–9.7] vs. 10.4 [10–11] g/dl, *P* < 0.001). There was no difference in 28-day mortality between the lower-threshold group compared with the higher-threshold group (hazard ratio, 0.94 [95%-CI, 0.59–1.48], *P* = 0.78). Within 28 days, 36.5% (95%-CI, 27.0–46.9) of the patients in the lower-threshold group compared with 39.5% (29.9–50.1) of the patients in the higher-threshold group had died. While there were no differences in ECMO-free, sedation-free, and organ dysfunction-free composites, the chance for successful weaning from mechanical ventilation within 28 days after ARDS onset was lower in the lower-threshold group (subdistribution hazard ratio, 0.36 [95%-CI, 0.15–0.86], *P* = 0.02).

**Conclusions:**

Transfusion at a hemoglobin concentration of 8 g/dl, as compared with a hemoglobin concentration of 10 g/dl, was not associated with an increase in 28-day mortality in adults with ARDS. However, a transfusion at a hemoglobin concentration of 8 g/dl was associated with a lower chance for successful weaning from the ventilator during the first 28 days after ARDS onset.

*Trial Registration*: ClinicalTrials.gov NCT03871166.

## Background

The acute respiratory distress syndrome (ARDS) is a common cause for hypoxemia in critically ill patients and associated with a high mortality [1, 2]. Despite advances in treatment strategies during recent decades such as the use of low tidal volume and low-pressure ventilation, adequate positive end-expiratory pressure (PEEP), and prone positioning, mortality of ARDS remains high, exceeding 60% for severe forms of ARDS [1, 3].

Besides cardiac output and pulmonary oxygen uptake, the hemoglobin concentration of the blood determines the blood oxygen carrying capacity and secures vital organ supply. To maintain a certain hemoglobin concentration during a patient’s stay on the intensive care unit (ICU), frequently the transfusion of packed red blood cells (RBCs) is required. For each individual blood transfusion, the transfusion-associated risks have to be carefully balanced against potential transfusion-associated benefits. In the recent decades, evidence has accumulated that accepting a lower than normal hemoglobin concentration can be safe in many different disease conditions and patient populations [4]. Therefore, current practice has gradually shifted to a more restrictive transfusion strategy in patients in the ICU [4–6].

Data for a target hemoglobin concentration and consecutive transfusion thresholds in patients with ARDS are poor. Current national and international guidelines on the management of patients with ARDS address this topic only vaguely or extrapolate recommendations for non-bleeding anemic, critically ill patients to patients with ARDS [6–9]. For ARDS patients treated with extracorporeal membrane oxygenation (ECMO), evidence is mainly based on case series and a recent large international survey demonstrated a high heterogeneity of the hemoglobin target concentration with most respondents using a higher transfusion threshold for patients on ECMO compared to other critically ill patients [10–13]. So far, due to the limited data addressing this issue, the efficacy and safety of different hemoglobin threshold for transfusion of RBCs in adults with ARDS are unknown.

Using a large cohort of 1044 patients with ARDS, the objective of this study was to assess the effect of a lower versus a higher transfusion threshold on short-term outcome in patients with ARDS. We hypothesized that using a transfusion threshold of a hemoglobin concentration of 8 g/dl was non-inferior compared to using a transfusion threshold of a hemoglobin concentration of 10 g/dl with regard to mortality and various failure-free days composites during the first 28 days after ARDS onset.

## Methods

### Study design and setting

This is a retrospective cohort study of ARDS patients who were admitted to the tertiary ARDS referral center of the Department of Anesthesiology and Intensive Care Medicine, Charité—Universitätsmedizin Berlin, Campus Virchow-Klinikum, Berlin, between January 2007 and December 2018, and who received transfusions of RBCs after ARDS onset. Patients were grouped according to their individual pre-transfusion hemoglobin concentration into five transfusion threshold groups. A lower-threshold group and a higher-threshold group were selected and matched to reduce selection bias and to ensure that basic assumptions to consider the two different hemoglobin thresholds as an intervention could be validated. Further details on basic assumptions are available from the Supplemental methods (Additional file 1). The two threshold groups were then compared on short-term outcome. The study was approved by the Medical Ethics Committee of Charité—Universitätsmedizin Berlin (No. EA1/018/19) and registered internationally (ClinicalTrials.gov NCT03871166).

### Participants

All adult patients fulfilling the criteria of the Berlin Definition [14] for ARDS were eligible for the study. Patients were excluded from analyses if they (1) were not transfused during the ARDS treatment, (2) received RBC transfusion only after 28 days after ARDS onset, (3) had other individual hemoglobin thresholds than 8 g/dl or 10 g/dl, (4) received veno-arterial ECMO, (5) died during the first 6 h after onset of ARDS, and (6) had incomplete data of prognostic determinants that were used for the matching procedure.

### Individual hemoglobin threshold and grouping

An individual hemoglobin threshold for RBC transfusion was calculated for each patient aiming at the hemoglobin threshold that was applied by the attending physicians during the 28-day period after ARDS onset. First, the lowest hemoglobin concentration during a period of 6 h prior to transfusion for each RBC unit during the 28-day period was identified. Then, the individual hemoglobin threshold of each patient was determined by averaging the lowest hemoglobin concentrations over the number of transfused RBC units. Further details are provided in the Supplemental methods (Additional file 1).

Patients were then grouped according to their individual hemoglobin threshold into five different transfusion threshold groups. According to clinical and methodological considerations, patients transfused at a hemoglobin concentration between 9 and 10 g/dl (higher-threshold group) and patients transfused at a hemoglobin threshold between 7 and 8 g/dl (lower-threshold group) were selected for analysis. Further details are provided in the Supplemental methods (Additional file 1). The coefficient of variation was used to confirm a low intra-patient variability of the individual hemoglobin threshold in the lower- and higher-threshold groups. The higher-threshold group was considered as the reference group with respect to primary and secondary endpoints. Subgroup analyses were performed for the cohort of patients with veno-venous ECMO and the cohort of patients without extracorporeal life support (ECLS).

### Endpoints

The primary endpoint was mortality within 28 days after ARDS onset. Secondary endpoints included mortality within 60 days after ARDS onset, ICU length of stay, variables of gas exchange and acid–base status, and “failure-free days” composites such as ECMO-free, ventilator-free (VFDs), sedation-free, organ dysfunction-free, renal replacement therapy-free, and vasopressor-free days. “Failure-free days” composites were defined and analyzed according to the most recent recommendations [15]. A detailed definition for each “failure-free days” composite is available from the Supplemental methods (Additional file 1).

### Data sources

All data required for this study were extracted from the electronic patient data management systems of the hospital. Further details are provided in the Supplemental methods (Additional file 1).

### Bias handling

When grouping a cohort of ARDS patients to two different hemoglobin thresholds, determinants introducing a selection bias should be identified and considered. Important prognostic determinants with regard to the study endpoints but not the study period were included into a matching procedure to reduce the effect of selection bias on study endpoints. Propensity score matching (PSM) was applied as a matching procedure to allow analysis of a non-randomized study in a way that mimics some of the particular characteristics of a randomized trial. The following prognostic determinants were included in the matching procedure: age, comorbidities (Charlson comorbidity index), ARDS severity (Berlin Definition), organ failure at ARDS onset (SOFA score, [16] pH, and lactate), prone positioning, need for ECLS (none, ECMO, extracorporeal lung assist [ECLA]), ECMO blood flow, ECMO sweep gas flow, PaO2:FiO2, driving pressure, and plateau pressure. Multivariable Cox proportional hazards regression was performed as complementary analysis on the primary endpoint including the same prognostic determinants as listed above. Further details on bias diagnostics and handling are available from the Supplemental methods (Additional file 1).

### Statistical analyses

Differences of continuous data were tested using the exact Mann–Whitney U test. Frequencies were tested using Fisher’s exact test. Differences of continuous data with respect to time were analyzed using nonparametric analysis for longitudinal data [17]. In PSM, the propensity score (PS) was estimated by fitting a logistic-regression model that included the prognostic determinants. Thereafter, a 1:1 pair matching was applied using the recommended method of nearest-neighbor matching without replacement, with a caliper width equal to 0.2 of the standard deviation of the logit of the PS [18]. The appropriateness of matching was assessed by comparing the standardized mean differences (SMD) of the prognostic determinants [18]. Further details on handling of imbalances in the matching procedure are available from the Supplemental methods (Additional file 1). Kaplan–Meier methods and Cox proportional hazards regression were used to compare mortality within 28 and 60 days after ARDS onset. The proportional hazard assumption was tested by scaled Schoenfeld residuals and by inspection of the hazard ratio (HR) plots. An equal distribution of censoring was checked. As recently recommended, the “failure-free days” composites were analyzed using a competing risk regression retaining (not censoring) patients experiencing the competing event (death) in the risk set [15]. The competing risk regression provides a subdistribution hazard ratio (SHR) that assesses the primary “net effect” size which is the chance of the lower-threshold group compared with the higher-threshold group for the particular event (ECMO removal, weaning from mechanical ventilation, stopping sedation, SOFA score < 6, stopping renal replacement therapy, stopping vasopressors) accounting for the existence of the alternative outcome of death. Cumulative incidence curves were presented for each “failure-free days” composite. Due to the exploratory study type, all analyses were considered to be non-confirmatory; a post hoc power analysis was omitted according to Hoenig and Heisey [19]. A two-tailed *p* value < 0.05 was considered statistically significant. The statistical analyses were performed with the use of R software, version 3.6.1 (R Project for Statistical Computing).

## Results

**Fig. 1:**
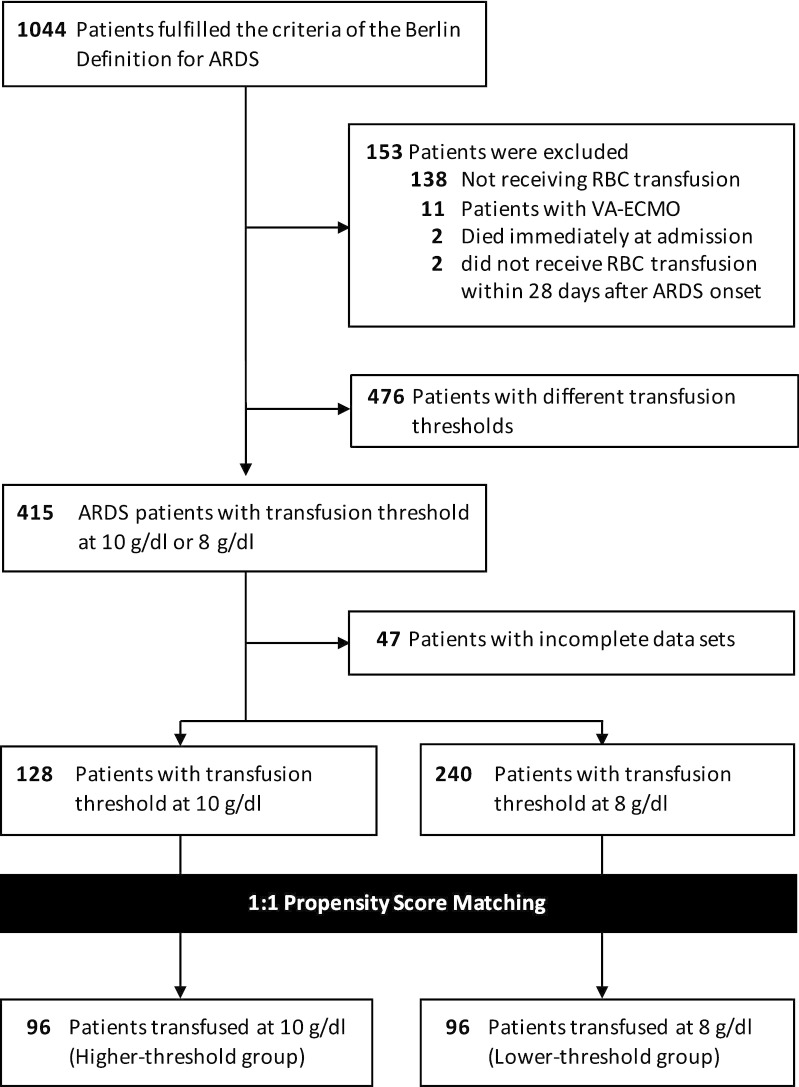
Study flow diagram. Patients were grouped according to their individual hemoglobin threshold into patients transfused at a hemoglobin concentration of 8 g/dl or less (lower threshold) and patients transfused at a hemoglobin concentration of 10 g/dl or less (higher threshold)

A total of 1044 patients with ARDS were identified. Of those, 153 patients were excluded because they did not receive RBC transfusions, received the first RBC transfusion later than 28 days after ARDS onset, died within 6 h after admission, or were treated with veno-arterial ECMO. For the remaining 891 patients (85.3% [95% CI, 83.0–87.4]), all transfused during the first 28 days after ARDS onset, a total of 54,915 hemoglobin concentrations (median 38 [IQR, 17–83]) were analyzed to calculate the patient-individual transfusion threshold. After exclusion of patients with other transfusion thresholds and patients with incomplete datasets, a total of 368 patients were included into the matching procedure. Applying a 1:1 paired PSM, 96 pairs (75% of the maximum possible pairs) were identified corresponding to a total of 192 matched patients (Fig. 1). In the matched cohort, the prognostic determinants were well balanced between the two hemoglobin threshold groups (PS 0.25 ± 0.16 vs. 0.27 ± 0.16, *P* = 0.63). The distribution of the PSs before and after matching is presented in Figures S2 and S3 (Additional file 1).

In the matched cohort, most patients had severe ARDS and the distribution of ARDS etiology was consistent with previously published cohorts. The majority of patients received prone positioning and a lung-protective ventilation with a high PEEP, a low tidal volume, and a low driving pressure. Baseline characteristics of the matched groups are shown in Table 1. There were no differences in demographic data, comorbidities, admissions scores, ARDS severity, ARDS etiology, need for ECLS, and ventilation and ECMO parameters.

**Table 1:** Baseline characteristics of the patients

Characteristic	Higher-threshold group (N = 96)	Lower-threshold group (N = 96)	*P* value	SMD
Age (years)	52.0 (37.8–61.0)	51.0 (38.0–61.0)	0.85	0.040
Male sex, n (%)	59 (61.5)	68 (70.8)	0.22	0.199
Body mass index (kg/cm)	27.3 (24.1–31.2)	26.3 (23.4–29.7)	0.27	0.152
Charlson comorbidity index	2.0 (1.0–4.0)	2.0 (0.0–5.0)	0.99	0.076
Immunocompromised, n (%)	22 (22.9)	22 (22.9)	0.99	< 0.001
Year of admission, n (%)			< 0.001	1.830
2007–2010	41 (42.7)	9 (9.4)		
2011–2014	46 (47.9)	14 (14.6)		
2015–2018	9 (9.4)	73 (76.0)		
SOFA at ARDS onset	12.0 (9.0–15.0)	12.0 (9.0–16.0)	0.80	0.022
SAPS II at ARDS onset	57.5 (38.0–69.2)	57.0 (41.8–68.0)	0.74	0.030
RASS at ARDS onset	− 5.0 (− 5.0 to − 4.0)	− 5.0 (− 5.0 to − 4.4)	0.49	< 0.001
Chronic lung disease, n (%)	25 (26.0)	26 (27.1)	0.99	0.024
Pulmonary origin, n (%)	78 (81.2)	77 (80.2)	0.99	0.026
Mechanical ventilation before admission (days)	1.0 (0.5–6.5)	1.5 (1.0–5.8)	0.59	0.004
ARDS severity, n (%)			0.99	< 0.001
Mild	0 (0)	0 (0)		
Moderate	10 (10.4)	10 (10.4)		
Severe	86 (89.6)	86 (89.6)		
ARDS etiology, n (%)			0.67	0.228
Pneumonia	64 (66.7)	57 (59.4)		
Aspiration	10 (10.4)	14 (14.6)		
Sepsis	7 (7.3)	6 (6.2)		
Pancreatitis	2 (2.1)	5 (5.2)		
Other	13 (13.5)	14 (14.6)		
Rescue therapy				
Inhaled nitric oxide, n (%)	80 (83.3)	62 (64.6)	0.005	0.437
Prone positioning, n (%)	72 (75.0)	67 (69.8)	0.52	0.117
Extracorporeal life support, n (%)			0.85	0.132
No ECLS	33 (34.4)	37 (38.5)		
ECLA	9 (9.4)	6 (6.2)		
ECMO	50 (52.1)	49 (51.0)		
Combined	4 (4.2)	4 (4.2)		
Ventilation parameters after initial optimization				
PaO_2_:FiO_2_ (mmHg)	129 (93–174)	130 (100–182)	0.65	0.050
Oxygenation index	17.5 (12.5–26.3)	17.9 (11.6–26.2)	0.70	0.082
PEEP (cm H_2_O)	16.0 (14.3–18.3)	18.0 (14.1–19.8)	0.23	0.035
Driving pressure (cm H_2_O)	16.0 (12.9–19.2)	16.0 (13.2–18.1)	0.87	0.009
Tidal volume (ml/kg PBW)	5.5 (3.8–7.1)	5.8 (4.1–6.9)	0.83	0.096
Compliance (ml/cm H2O)	27 (19.0–36.4)	30 (20.7–38.2)	0.41	0.028
ECMO initiation (ICU day)	0 (0–0)	0 (0–0)	0.55	0.050
ECMO pump flow (l/min)	3.8 (3.0–4.3)	3.8 (3.2–4.2)	0.88	0.090
ECMO sweep gas flow (l/min)	4.0 (3.0–6.8)	4.0 (3.0–6.0)	0.44	0.156
Septic shock, n (%)	53 (55.2)	47 (50.5)	0.56	0.094
Lactate (mg/dl)	19.0 (13.0–43.5)	19.0 (11.8–56.8)	0.95	0.007
pH	7.3 (7.2–7.4)	7.3 (7.2–7.3)	0.98	0.019
RRT, n (%)	60 (62.5)	63 (65.6)	0.76	0.065

Data are expressed as median [25%, 75% quartiles] or frequencies [%], as appropriate. *P* values were calculated using the exact Wilcoxon–Mann–Whitney test and the Fisher’s exact test, as appropriate. Standardized mean differences (SMD) are provided

*SOFA* Sequential Organ Failure Assessment, *SAPS* Simplified Acute Physiology Score, *RASS* Richmond Agitation-Sedation Scale, *ECLS* extracorporeal life support, *ECLA* pumpless extracorporeal lung assist, *ECMO* extracorporeal membrane oxygenation, *PEEP* positive end-expiratory pressure, *PBW* predicted body weight, *ICU* intensive care unit, *RRT* renal replacement therapy

### Hemoglobin concentrations and transfusion

**Fig. 2:**
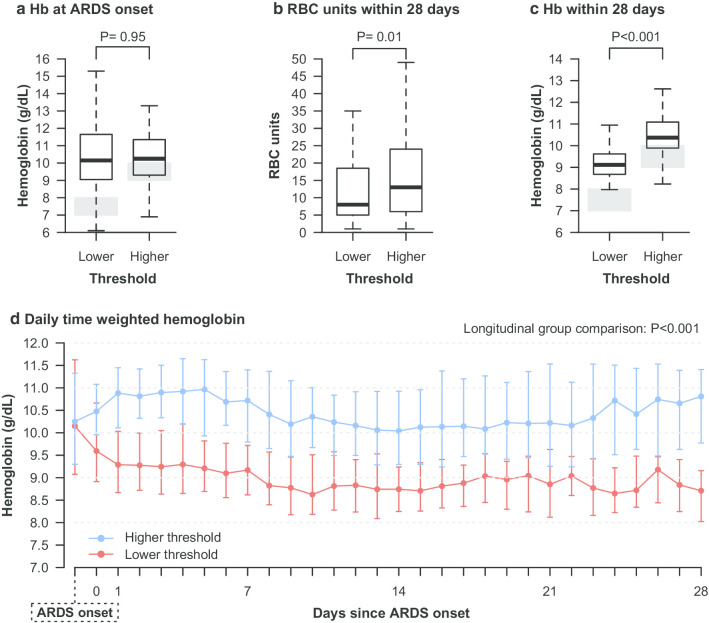
Hemoglobin concentrations and transfusion requirements between the lower-threshold group and higher-threshold group. The hemoglobin concentrations at ARDS onset (**a**), the number of transfused RBC units within 28 days of ARDS therapy (**b**), and the hemoglobin concentrations within 28 days of ARDS therapy (**c**) are presented. Gray boxes help to visualize the coherence of the range of individual hemoglobin thresholds that was used for grouping and the hemoglobin concentrations at admission and within 28 days of ARDS therapy. Median daily time-weighted average hemoglobin concentrations during 28 days of ARDS therapy in the lower-threshold group and higher-threshold group (**d**). Daily time-weighted average hemoglobin concentrations overcome the complexity that number and timing of daily blood gas samples were not exactly the same in all patients. First values were the baseline hemoglobin concentrations at onset of ARDS. Day 0 was defined as the time of ARDS onset to the end of that day. Data are shown as median and 25th and 75th percentiles

The median individual hemoglobin threshold in the lower-threshold group was 7.5 g/dl (IQR, 7.3–7.8) compared to 9.4 g/dl (9.2–9.7) in the higher-threshold group with a low intra-patient variability in both groups (8% [IQR, 5–10] and 10% [7–12], respectively). To confirm that hemoglobin thresholds could be considered as an intervention in our cohort study, the hemoglobin concentrations at ARDS onset and within 28 days of ARDS therapy, and the number of transfused RBC units were compared. The median hemoglobin concentration at ARDS onset was similar between the lower and the higher-threshold group (10.1 g/dl [9.1–11.6] vs. 10.2 g/dl [9.3–11.3], *P* = 0.95) (Fig. 2a). The median number of transfused RBC units (8 units [5–18] vs. 13 units [6–24], *P* = 0.01) and the median hemoglobin concentration within 28 days of ARDS therapy (9.1 g/dl [8.7–9.7] vs. 10.4 g/dl [9.9–11.1], *P* < 0.001) were lower in the lower-threshold group compared to higher-threshold group (Fig. 2b, c). The time-weighted average hemoglobin concentrations during 28 days of ARDS therapy indicated that hemoglobin levels were above the respective transfusion threshold in each threshold group and that a steady difference in average hemoglobin concentration of at least 1 g/dl between the two threshold groups was present for 28 days of ARDS therapy (Fig. 2d). In this respect, daily transfusion requirements were highest during the first days after ARDS onset (Figure S4, Additional file 1). Furthermore, the percentage of single unit transfusions was higher in the lower-threshold group, compared to the higher-threshold group (45.9% [43.6–48.3] vs. 34.9% [32.3–37.7], *P* < 0.001).

### Endpoints

**Fig. 3:**
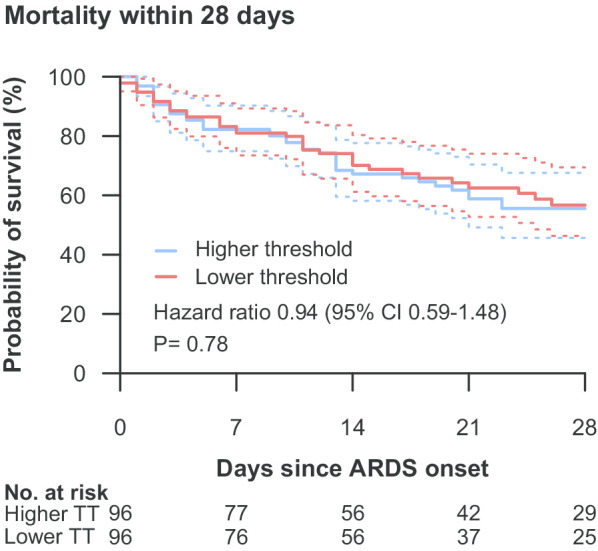
Kaplan–Meier survival estimates of the mortality within 28 days after onset of ARDS between the lower-threshold group and higher-threshold group. For each curve, 95% confidence intervals (dotted lines) are shown. The hazard ratio with 95% confidence intervals are provided. The median observation time was 24 days (IQR, 13–28) in the lower-threshold group and 27 days (17–28) in the higher-threshold group. There was no difference in censoring between the two transfused groups (*P* = 0.30)

There was no difference in 28-day mortality between the lower-threshold group compared with the higher-threshold group (hazard ratio [HR], 0.94 [95%-CI, 0.59–1.48], *P* = 0.78) (Fig. 3). The median observation time was 24 days (IQR, 13–28) in the lower-threshold group and 27 days [17–28] in the higher-threshold group. There was no difference in censoring between the two transfused groups (*P* = 0.30). Within 28 days, 36.5% (95%-CI, 27.0–46.9) of the patients in the lower-threshold group compared with 39.5% (29.9–50.1) of the patients in the higher-threshold group had died. The complementary analysis using multivariable Cox proportional hazards regression (n = 368 patients) confirmed that there was no difference in 28-day mortality between the lower-threshold group compared with the higher-threshold group (HR, 0.86 [0.56–1.29], *P* = 0.46).

Furthermore, there was no difference in 60-day mortality between the two threshold groups (HR, 0.95 [0.61–1.47], *P* = 0.82). Within 60 days, 40.6% (95%-CI, 30.8–51.1) of the patients in the lower-threshold group compared with 43.7% (33.7–54.2) of the patients in the higher-threshold group had died. Median ICU length of stay was not different between the lower-threshold and the higher-threshold group (16 days [9–27] vs. 17 days [9–30], *P* = 0.77).

**Fig. 4:**
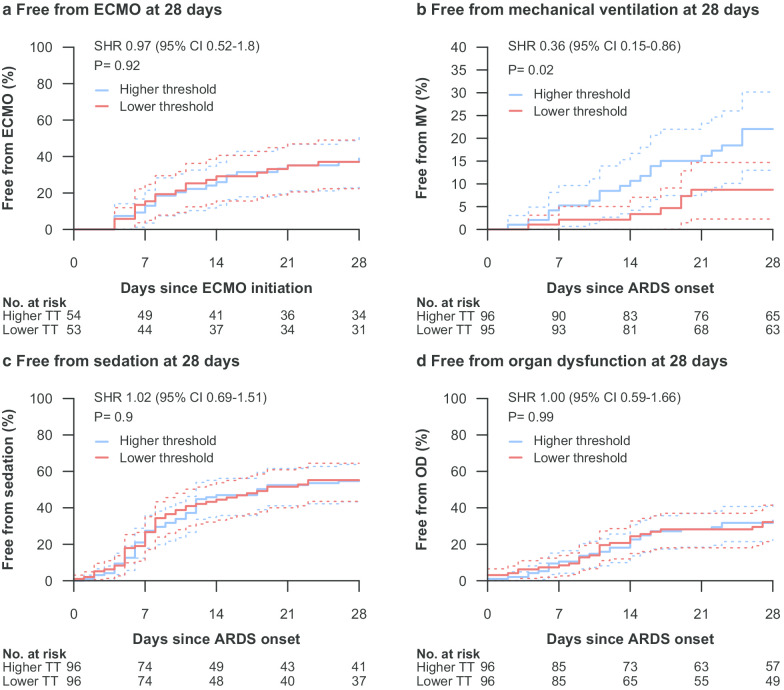
Cumulative incidence curves of ECMO-free (**a**), ventilator-free (**b**), sedation-free (**c**), and organ dysfunction-free (**d**) days composites between the lower-threshold group and higher-threshold group. For each curve, 95% confidence intervals (dotted lines) are shown. The subdistribution hazard ratio (SHR) with 95% confidence intervals is provided. The SHR is calculated from a competing risk regression providing the chance of the lower-threshold group compared with the higher-threshold group for the particular event (ECMO removal, weaning from mechanical ventilation, RASS 0 or − 1, SOFA score < 6) accounting for the existence of the alternative outcome of death. For better interpretation, the y-axis of the ventilator-free days composite (**b**) is scaled from 0 to 40% instead of 0 to 100%. Definition of abbreviations: *TT*transfusion threshold, *ECMO* extracorporeal membrane oxygenation, *MV* mechanical ventilation, *OD *organ dysfunction

ECMO-free (subdistribution hazard ratio [SHR], 0.97 [95%-CI, 0.52–1.80], *P* = 0.92), sedation-free (SHR, 1.02 [0.69–1.51], *P* = 0.90), organ dysfunction-free (SHR, 1.00 [0.59–1.66], *P* = 0.99), renal replacement therapy-free (SHR, 1.11 [0.58–2.11], *P* = 0.76), and vasopressor-free (SHR, 1.09 [0.70–1.69], *P* = 0.72) days composites were similar between the lower- and higher-threshold groups (Figure S5, Additional file 1). In contrast, patients in the lower-threshold group compared to patients in the higher-threshold group had a significantly lower chance for weaning from mechanical ventilation within 28 days after ARDS onset (SHR of VFDs composite, 0.36 [0.15–0.86], *P* = 0.02) (Fig. 4b). Within 28 days, 7.3% (95%-CI, 3.2–15.0) of the patients in the lower-threshold group compared with 20.8% (13.4–30.5) of the patients in the higher-threshold group were successfully weaned from mechanical ventilation.

Determinants of gas exchange and acid–base status did not differ between the lower- and higher-threshold groups within 28 days after ARDS onset (Figure S6, Additional file 1).

### Subgroup analyses

The subgroup analyses consisted of 99 patients with veno-venous ECMO and 70 patients without extracorporeal life support (ECLS). There was no difference in 28-day mortality between the lower-threshold group compared with the higher-threshold group in patients treated with ECMO (HR, 1.19 [95%-CI, 0.67–2.13], *P* = 0.55), or in patients treated without ECLS (HR, 0.64 [0.26–1.57], *P* = 0.33).

**Fig. 5:**
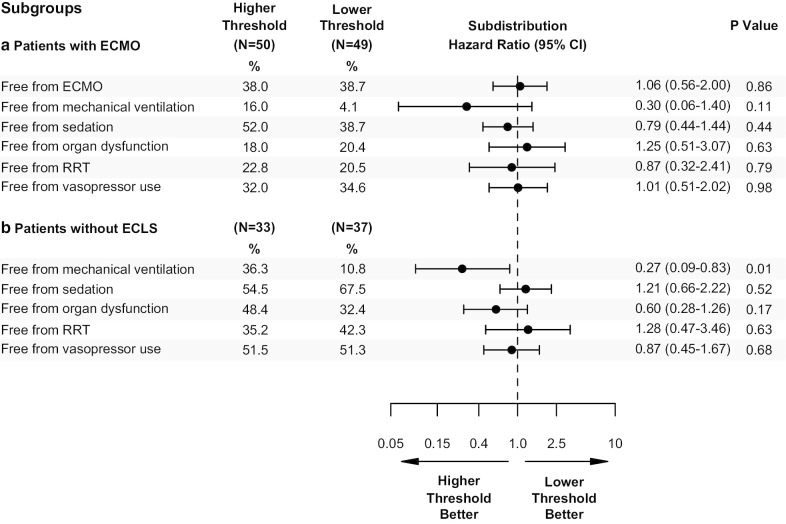
Subgroup analyses of failure-free composites between the lower-threshold group and higher-threshold group in the cohort of patients with ECMO (**a**) and the cohort of patients without ECLS (**b**). For each failure-free composite, the cumulative events within 28 days after ARDS onset and the appropriate effect measure (subdistribution hazard ratio [SHR]) with 95% confidence intervals is provided. The SHR has to be interpreted as a chance of the lower-threshold group compared with the higher-threshold group for reaching the particular event (ECMO removal, weaning from mechanical ventilation, stopping sedation, SOFA score < 6, stopping renal replacement therapy, stopping vasopressors) accounting for the existence of the alternative outcome of death. The subdistribution hazard ratio is presented on a log-transformed axis. Definition of abbreviations: *RRT* renal replacement therapy, *ECMO* extracorporeal membrane oxygenation, *ECLS* extracorporeal life support

ECMO-free, sedation-free, organ dysfunction-free, renal replacement therapy-free, and vasopressor-free days composites were similar between the lower- and higher-threshold groups in both, ECMO patients and patients without ECLS (Fig. 5). Among patients without ECLS, patients in the lower-threshold group compared to patients in the higher-threshold group had a significantly lower chance for weaning from mechanical ventilation within 28 days after ARDS onset (SHR of VFDs composite, 0.27 [0.09–0.83], *P* = 0.01) (Fig. 5b). Within 28 days, 10.8% (95%-CI, 3.5–26.3) of the patients in the lower-threshold group compared with 36.3% (20.9–54.8) of the patients in the higher-threshold group were successfully weaned from mechanical ventilation.

## Discussion

In this study, patients were grouped according to their individual hemoglobin concentration that reflected the hemoglobin threshold for RBC transfusion applied during the 28-day period after ARDS onset. Our results suggest that in patients with ARDS who received a blood transfusion 28-day mortality was not higher when a hemoglobin concentration of 8 g/dl was used as transfusion threshold compared to a hemoglobin concentration of 10 g/dl. However, a lower transfusion threshold was associated with a lower chance for successful weaning from the ventilator during the first 28 days after ARDS onset.

In recent years, the number of randomized controlled trials (RCTs) comparing restrictive transfusion thresholds with liberal transfusion thresholds has considerably increased with now more than 30 large RCTs [4]. Classically, hemoglobin levels of 7–8 g/dl are considered as restrictive and hemoglobin levels of 9–10 g/d as liberal transfusion thresholds [4]. In summary, data of these RCTs showed that a restrictive transfusion threshold was not inferior compared to a liberal transfusion threshold with regard to mortality and adverse effects [4]. Current evidence-based recommendations propose a transfusion threshold at a hemoglobin concentration of 7 g/dl for hemodynamically stable ICU patients and patients with septic shock [6, 20]. However, for certain subgroups such as patients with hip fractures and cardiovascular diseases higher hemoglobin thresholds are recommended [6, 20]. For patients with ischemic heart disease, or brain injury, data are not sufficient to formulate reliable recommendations. Likewise, although six RCTs examined transfusion strategies in ICU-patients, these results cannot simply be extrapolated to the specific subgroup of patients with ARDS [21–26]. While blood oxygen carrying capacity is determined by cardiac output, hemoglobin concentration, and pulmonary gas exchange, the latter is severely compromised in patients with ARDS. The blood oxygen carrying capacity in the complex physiology of this patient population needs to be considered when evaluating the optimal hemoglobin concentration to optimize clinical outcome. Conduction of interventional studies in patients with ARDS, often including therapy with ECMO, is challenging because of the complexity of the disease, the frequent inability to obtain timely patient consent for interventions and the concomitant long recruiting phase [27–29]. Therefore, using a propensity score based matching procedure is particularly helpful to investigate different transfusion thresholds in this challenging patient population, especially if multiple endpoints are addressed.

In accordance with the results of previous RCTs on transfusion thresholds in various patient populations, the data of this study demonstrate that a lower (restrictive) transfusion threshold compared to a higher (liberal) transfusion threshold was not associated with an increase in 28-day mortality in ICU-patients with ARDS who received a blood transfusion [4, 22, 24, 26, 30]. In addition, applying a higher transfusion threshold was not associated with an increase in the chances for a successful weaning from ECMO within 28 days after onset of ARDS. Therefore, these data argue against the need of a higher transfusion threshold in ARDS patients treated with veno-venous ECMO [6, 10]. In particular, in cases without evidence for anemic hypoxia as reflected by lactate levels and ECMO blood flow settings, a lower transfusion threshold can be safe. Nevertheless, comparing a restrictive versus a liberal transfusion strategy in patients treated with ECMO remains a research priority for the upcoming years [6]. In contrast to the weaning from ECMO, chances for a successful extubation or weaning from the ventilator after tracheostomy within 28 days after onset of ARDS were lower when a lower transfusion threshold was applied. Apart from gas transport and gas exchange disorders, low hemoglobin concentrations can be an important determinant for weaning failure because anemia leads to compensatory stress of the cardio-respiratory system. In patients with a chronic impairment of the respiratory system, RBC transfusion leads to a significant reduction of minute ventilation and work of breathing [31, 32]. In a retrospective analysis of difficult-to-wean patients, patients with a hemoglobin concentration of 8–10 g/dl were more often successfully weaned from mechanical ventilation compared to patients whose hemoglobin concentration was below 8 g/dl [33]. So far, in the absence of any prospective studies investigating effects and complications of different transfusion thresholds in patients undergoing a prolonged pulmonary weaning, our data suggest that a higher hemoglobin threshold in ARDS patients who receive a blood transfusion might facilitate a successful extubation or weaning from mechanical ventilation.

This study has several limitations. Grouping a cohort of ARDS patients according to different hemoglobin thresholds introduced a selection bias with respect to prognostic determinants and study periods between the threshold groups. Furthermore, the studied cohort included mainly patients admitted with a severe ARDS and a concomitant high rate of patients receiving therapy with veno-venous ECMO [1, 28]. Although we cannot rule out that further unknown confounders have affected the results, the selection bias of prognostic determinants in the matched cohort could be reduced to a minimum as indicated by the low standardized mean differences for the majority of variables. Furthermore, we could demonstrate that the primary endpoint was not systematically affected by the change of the transfusion practice during the study period in this patient cohort. Due to the retrospective study design, the individual hemoglobin threshold had to be calculated for each individual patient, allowing less precision than in a prospective study. However, the low coefficient of variation in each transfusion threshold group confirmed a low intra-patient variability of the individual hemoglobin thresholds. Furthermore, the calculation of the individual hemoglobin threshold resulted in the exclusion of 138 patients (13%) that were not transfused. Due to the retrospective study design, transfusion thresholds could only be determined in patients that had received a blood transfusion during the observation period. Patients, who did not receive any RBC transfusion because a lower transfusion threshold was applied, were therefore missed in the analysis. Due to the retrospective study design, evaluation whether a lower transfusion threshold resulted in a lower transfusion rate in the restrictive group was not possible. Despite an appropriate PSM, the current study cannot mimic a RCT, and therefore, comparison of the results of the current study with results of studies that investigated different transfusion thresholds in a prospective setting has to be interpreted with caution. The hemoglobin increment after RBC transfusion is inversely proportional to the pre-transfusion hemoglobin level [34]. This might explain hemoglobin levels around 9 g/dl for patients transfused at a hemoglobin threshold between 7 and 8 g/dl. Although the analyses of this study have to be interpreted as non-confirmatory, Kaplan–Meier curves of the primary endpoint were nearly identical. Therefore, it is unlikely that the loss of statistical power, e.g., due to incomplete datasets and excluded patients, debilitated the validity to detect a significant difference in 28-day mortality between patients in the lower and the higher transfusion threshold groups.

## Conclusions

This is the first study exclusively comparing two hemoglobin thresholds for RBC transfusion in patients with ARDS who received a blood transfusion. This single-center cohort study carefully indicates that transfusion at a hemoglobin threshold of 8 g/dl, as compared with a hemoglobin threshold of 10 g/dl, was not associated with an increase in 28-day mortality in adults with ARDS who received a blood transfusion. Whether a higher hemoglobin concentration facilitates successful weaning from the ventilator should be subject of further research.

## Supplementary Information

**Additional file 1.** Supplemental material including supplemental Methods, Table, and Figures.

## Data Availability

The datasets used and/or analyzed during the current study are available from the corresponding author on reasonable request.

## Abbreviations

ARDS: Acute respiratory distress syndrome
ECLA: Extracorporeal lung assist
ECLS: Extracorporeal life support
ECMO: Extracorporeal membrane oxygenation
HR: Hazard ratio
ICU: Intensive care unit
MV: Mechanical ventilation
OD: Organ dysfunction
PEEP: Positive end-expiratory pressure
PSM: Propensity score matching
RASS: Richmond Agitation-Sedation Scale
RBCs: Red blood cells
RCTs: Randomized controlled trials
RRT: Renal replacement therapy
SAPS: Simplified Acute Physiology Score
SHR: Subdistribution hazard ratio
SMD: Standardized mean differences
SOFA: Sequential Organ Failure Assessment
TT: Transfusion threshold
VFDs: Ventilator-free days
