# Supplementary material for: Lower versus higher hemoglobin threshold for transfusion in ARDS patients with and without ECMO
Source: Crit Care. 2020 Dec 16;24:697. doi: 10.1186/s13054-020-03405-4 (PMC7740070; doi:10.1186/s13054-020-03405-4)
Supplement: Supplementary file 1 — Additional file 1. Supplemental material including supplemental Methods, Table, and Figures. [file 13054_2020_3405_MOESM1_ESM.pdf]

Additional file 1

Lower versus Higher Hemoglobin Threshold for Transfusion in ARDS Patients with and without ECMO

O. Hunsicker, L. Materne, V. Bünger, A. Krannich, F. Balzer, C. Spies, R. C. Francis, S. Weber-Carstens, M. Menk, J. A. Graw

Table of Contents

Supplemental Methods 2
Hemoglobin threshold as an intervention 2
Individual hemoglobin threshold and grouping 3
Extended flow diagram: 5
Definition of "failure-free days" composites 6
ECMO-free days composite 6
Ventilator-free days composite (VFDs) 6
Sedation-free days composite 7
Organ dysfunction-free days composite 7
Vasopressor-free days composite 7
Renal replacement therapy-free days composite 8
Bias handling 8
Data sources 10
Supplemental Tables 11
Table S1: Baseline characteristics of the unmatched cohort. 11
Supplemental Figures 13
Figure S1: Transfusion practice and mortality during the study period. 13
Figure S2: Distribution of propensity scores. 14
Figure S3: Distribution of propensity scores – detailed visualization. 15
Figure S4: Daily transfusion requirements. 16
Figure S5: Additional failure-free composites. 17
Figure S6: Determinants of gas exchange and acid-base status. 18
References 19

Supplemental Methods

Hemoglobin threshold as an intervention

Investigating hemoglobin thresholds in non-randomized cohort studies is complex because three basic assumptions need to be fulfilled before the different hemoglobin thresholds could be considered as a valid intervention. First, the baseline hemoglobin concentration at the time of ARDS onset should be similar between the two threshold groups. Second, the transfusion requirements should be greater in the higher-threshold group. Finally, there should be a significant and clinically relevant difference in hemoglobin concentration between the threshold groups.

In non-randomized cohort studies, it must be ensured that these basic assumptions can be reviewed and confirmed even after controlling for between-group differences. Using regression analyses is not appropriate in this case because diagnostic outputs do neither allow to determine the degree to which the fitted regression model has successfully eliminated differences in baseline hemoglobin concentrations between the both threshold groups, nor allow to validate differences in transfusion requirements and hemoglobin concentrations. Therefore, in our study, a matching procedure was performed to control for between-group differences while being able to review and confirm the basic assumptions in the matched cohort.

To demonstrate that there was a steady clinically relevant difference in hemoglobin concentration between the threshold groups during the 28-day period after ARDS onset, daily time-weighted average hemoglobin concentrations were calculated for this period. Time-weighted average hemoglobin concentrations were calculated in accordance with the work of Finney et al. [1] and overcome the complexity that number and timing of daily blood gas samples were not exactly the same in all patients. For each day during the 28-day period all measured hemoglobin values and their corresponding sampling time were considered for the calculations. The time-weighted average of hemoglobin concentration was calculated assuming a linear trend between two consecutive hemoglobin measurements and giving a time value to such

measurements. For example, a hemoglobin concentration of 10 g/dl at 10 am followed by 6 g/dl at 2 pm, yields a value of 8 g/dl weighted by 4 hours. For each day the sum of such weighted values was then divided by total hours of observation (24 hours, except for the first day depending on time of admission) to calculate the daily time-weighted average hemoglobin concentration for each patient.

Individual hemoglobin threshold and grouping

An individual hemoglobin threshold for RBC transfusion was assessed for each patient aiming at the hemoglobin threshold that was applied by the attending physicians during the 28-day period after ARDS onset. First, the lowest hemoglobin concentration during a period of 6 hours prior to transfusion for each RBC unit during the 28-day period was identified. Then, the individual hemoglobin threshold of each patient was determined by averaging the lowest hemoglobin concentrations over the number of transfused RBC units. In this retrospective cohort study, only transfusion data after ARDS onset and admittance to the tertiary ARDS referral center was included. In a retrospective study setting with an absence of an a priori defined and documented transfusion threshold this approach allowed the clearest and best determination of the individual transfusion threshold for each individual patient. A putative transfusion threshold can reliably be identified even if two consecutive RBC units (as a formerly accepted transfusion practice) were transfused by ensuring that the lowest hemoglobin concentration that indicated the first RBC unit was most probably also used for the second RBC unit. Furthermore, the correct hemoglobin concentration leading to the decision to transfuse could most likely be identified even if transfusion was delayed after indication.

Following identification of the individual transfusion threshold, patients were grouped into five different transfusion threshold groups. According to clinical and methodological considerations, patients transfused at a hemoglobin concentration of 10 g/dl or less, but higher than 9 g/dl (higher-threshold group) and patients transfused at a hemoglobin threshold of 8 g/dl or less, but

higher than 7 g/dl (lower-threshold group) were selected for analysis (see extended flow diagram – next page).

Patients with a hemoglobin level greater than 10 g/dl as transfusion threshold were excluded because none of the current guidelines, recommendations or metaanalysis sees an indication to transfuse patients with a hemoglobin level greater than 10 g/dl [2-5]. Furthermore, patients with a hemoglobin level lower than 7 g/dl as transfusion threshold were excluded because this group showed a great inhomogeneity of disease severity, a high intra-patient variability for the transfusion threshold, and a failure to fulfill basic assumptions after matching and therefore could not be considered as a lower-threshold group. To guarantee that the two remaining transfusion threshold groups that were selected for the analysis showed a difference of at least 1 g/dl for their transfusion threshold, it was necessary to exclude the group with a transfusion threshold of a hemoglobin level between 8 g/dl and 9 g/dl. The coefficient of variation was used to confirm a low intra-patient variability of the individual hemoglobin thresholds. The higher threshold group was considered as the reference group with respect to primary and secondary endpoints. Subgroup analyses were performed in the cohort of patients with veno-venous ECMO and the cohort of patients without extracorporeal life support (ECLS).

Extended flow diagram:

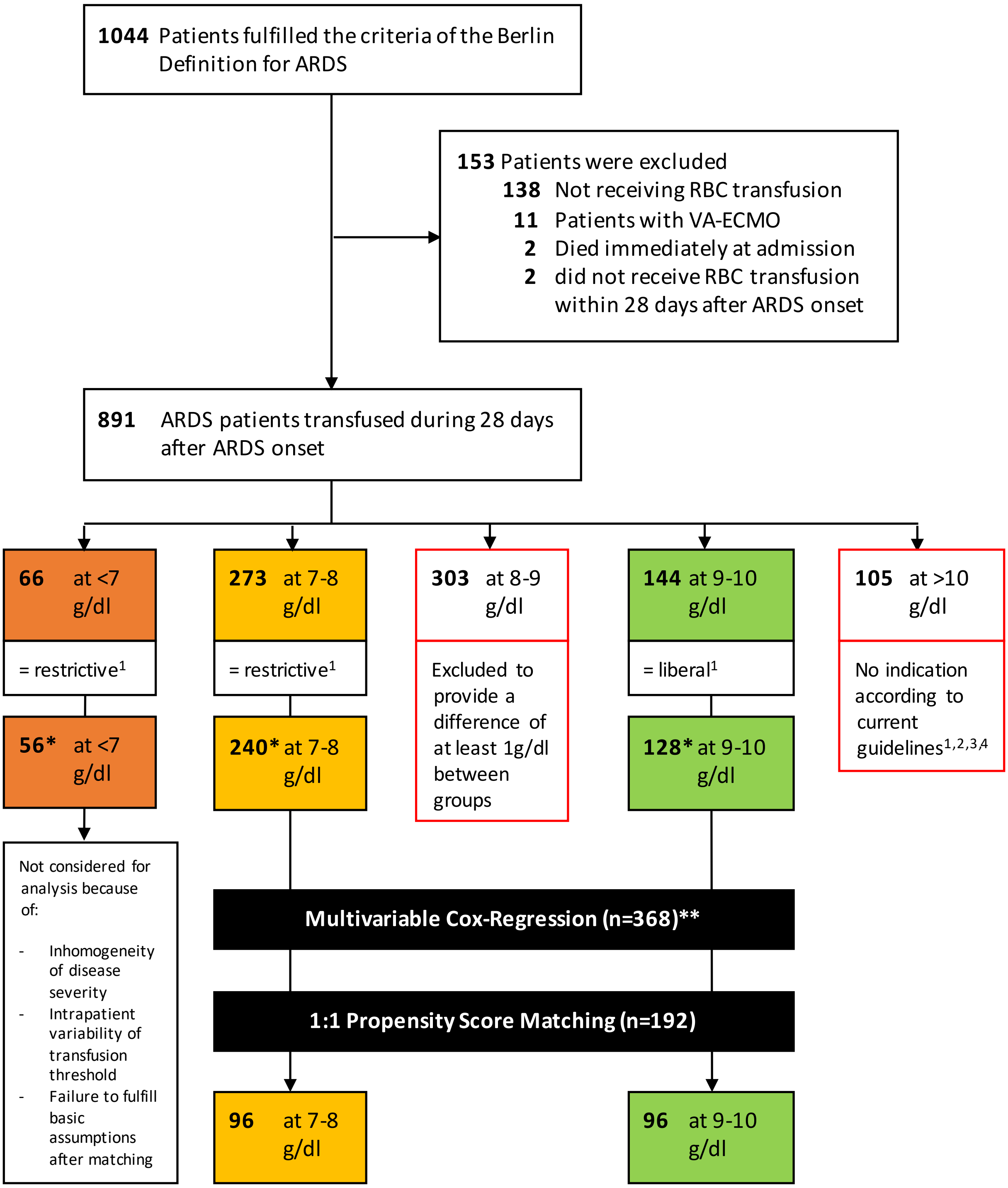

Legend:

* **After excluding patients with incomplete data sets.**

** **Multivariable Cox proportional hazards regression was performed as complementary analysis on the primary endpoint.**

References:

1. Carson JL, Stanworth SJ, Roubinian N, Fergusson DA, Triulzi D, Doree C, Hebert PC. **Transfusion thresholds and other strategies for guiding allogeneic red blood cell transfusion**. Cochrane Database Syst Rev 2016; 10: CD002042.
2. Carson JL, Guyatt G, Heddle NM, Grossman BJ, Cohn CS, Fung MK, Gernsheimer T, Holcomb JB, Kaplan LJ, Katz LM et al: **Clinical Practice Guidelines From the AABB: Red Blood Cell Transfusion Thresholds and Storage.** *JAMA : the journal of the American Medical Association* 2016, 316(19):2025-2035.
3. Vlaar AP, Oczkowski S, de Bruin S, Wijnberge M, Antonelli M, Aubron C, Aries P, Duranteau J, Juffermans NP, Meier J, Murphy GJ, Abbasciano R, Muller M, Shah A, Perner A, Rygaard S, Walsh TS, Guyatt G, Dionne JC, Cecconi M: **Transfusion strategies in non-bleeding critically ill adults: a clinical practice guideline from the European Society of Intensive Care Medicine.** *Intensive Care Med* 2020.
4. Mueller MM, Van Remoortel H, Meybohm P, Aranko K, Aubron C, Burger R, Carson JL, Cichutek K, De Buck E, Devine D, Fergusson D, Follea G, French C, Frey KP, Gammon R, Levy JH, Murphy MF, Ozier Y, Pavenski K, So-Osman C, Tiberghien P, Volmink J, Waters JH, Wood EM, Seifried E, Group IPF. **Patient Blood Management: Recommendations From the 2018 Frankfurt Consensus Conference.** JAMA 2019; 321: 983-997.

Definition of "failure-free days" composites

"Failure-free days" composites were assessed defined and analyzed according to the most recent recommendations [6]. Briefly, except for the ECMO-free days composite, we defined the ARDS onset as start time, used a timeframe of 28 days, assigned all 28-day non-survivors 0 failure-free days irrespective if the event of interest occurred before, and censored observations after 28 days. In the following a detailed definition of each "failure-free days" composite is presented.

ECMO-free days composite

Start time	Day of ECMO initiation.
Timeframe	28 days.
Successful ECMO removal	ECMO removal without re-initiation up to day 28.
Interval ECMO removal	We counted from the day of final ECMO removal if there were more than one ECMO initiation in the first 28 days.
Value for decedent after successful ECMO removal	All 28-day non-survivors were counted 0 ECMO-free days, irrespective if the ECMO could have been removed successfully, and censored observations after 28 days.

Ventilator-free days composite (VFDs)

The ventilator-free days composite was defined according to the most recent recommendations by Yehya et al [6].

Start time	Day of ARDS onset.
Timeframe	28 days.
Successful weaning from mechanical ventilation	Extubation >48 hours without reintubation or >48 hours off of positive pressure in patients with tracheostomy.
Interval extubation	We counted from the day of final successful extubation if there were repeat intubation episodes in the first 28 days.
Non-invasive support and tracheostomies	Non-invasive support was not counted and tracheostomies were treated as other invasive ventilation.
Value for extubated decedent	All 28-day non-survivors were counted 0 VFDs, irrespective of their intubation status, and censored observations after 28 days.

Sedation-free days composite

Start time	Day of ARDS onset.
Timeframe	28 days.
Successful weaning from sedation	Richmond Agitation-Sedation Scale (RASS) 0 or -1. [Successful weaning from sedation was defined as published previously [7, 8].]
Interval weaning from sedation	We counted from the day of final RASS 0 or -1 if there were repeat episodes of need for a deeper sedation in the first 28 days.
Value for decedent after successful weaning from sedation	All 28-day non-survivors were counted 0 sedation-free days, irrespective of successful weaning from sedation before and censored observations after 28 days.

Organ dysfunction-free days composite

Start time	Day of ARDS onset.
Timeframe	28 days.
Successful recovery from organ dysfunction	Sequential Organ Failure Assessment (SOFA) score <6 [Recovery from organ dysfunction was defined as published previously [9].]
Interval recovery from organ dysfunction	We counted from the day of final recovery from organ dysfunction if there were repeat episodes of recurrence of organ dysfunction in the first 28 days.
Value for decedent after recovered organ function	All 28-day non-survivors were counted 0 organ dysfunction-free days, irrespective of successful recovery from organ dysfunction before and censored observations after 28 days.

Vasopressor-free days composite

Start time	Day of ARDS onset.
Timeframe	28 days.
Successful weaning from vasopressors	Stopping vasopressors without re-initiation up to day 28.

Interval weaning from vasopressors	We counted from the day of finally stopping vasopressors if there were repeat episodes of starting vasopressors again in the first 28 days.
Value for decedent after weaning from vasopressors	All 28-day non-survivors were counted 0 vasopressor-free days, irrespective of successful weaning from vasopressors before and censored observations after 28 days.

Renal replacement therapy-free days composite

Start time	Day of ARDS onset.
Timeframe	28 days.
Successful weaning from renal replacement therapy	Stopping renal replacement therapy without re-initiation up to day 28.
Interval weaning from renal replacement therapy	We counted from the day of finally stopping renal replacement therapy if there were repeat episodes of re-initiation of renal replacement therapy in the first 28 days.
Value for decedent after weaning from renal replacement therapy	All 28-day non-survivors were counted 0 renal replacement therapy-free days, irrespective of successful weaning from renal replacement therapy before and censored observations after 28 days.

Bias handling

When grouping a cohort of ARDS patients to two different hemoglobin thresholds, two main determinants introducing a selection bias should be considered. First, the circumstance that a higher disease severity of a patient might have motivated the attending physicians to trigger higher hemoglobin thresholds for transfusion to increase oxygen delivery capacity. An inhomogeneity between the two threshold groups with respect to ARDS severity, the need for rescue therapies such as ECLS, and ventilation and ECMO parameters indicated that the latter is at least partly true for our cohort of ARDS patients (Table S1). Second, the transfusion practice might have changed during the study period towards a more restrictive transfusion practice with lower hemoglobin thresholds. The latter might be relevant if both the hemoglobin thresholds and the mortality decrease over the study period. While we observed a significant decrease of

hemoglobin threshold, there was no significant decrease in mortality during the study period (Figure S1 A, B). Therefore, important prognostic determinants with regard to the study endpoints, but not the study period were included into a matching procedure to reduce the effect of the selection bias on study endpoints. A propensity score matching (PSM) was applied as a matching procedure. PSM allows the analysis of a non-randomized study in a way that it mimics some of the particular characteristics of a randomized trial. Randomization is mimicked by optimally balancing propensity scores (PSs), which is the probability of treatment assignment conditional on the confounding variables. Given the current evidence on prognostic baseline determinants of outcome in critical ill patients with ARDS, the following confounding variables were included into the matching procedure: age, comorbidities (Charlson comorbidity index), ARDS severity (Berlin Definition), organ failure at ARDS onset (SOFA score [10], pH, and lactate), prone positioning, need for ECLS (none, ECMO, extracorporeal lung assist [ECLA]), ECMO blood flow, ECMO sweep gas flow, PaO2:FiO2, driving pressure, and plateau pressure. Using baseline characteristics of the patients for the propensity score matching was considered to be appropriate because daily transfusion requirements indicated that the majority of RBC units was given during the first days of the 28-day period of the ARDS treatment (Figure S3). In addition, the hemoglobin concentration at ARDS onset was included to ensure a similar distribution between the two threshold groups.

The fitting of the matching procedure was assessed by comparing the standardized mean differences (SMD) of the prognostic determinants. A generally accepted criterion for a threshold of the SMD that is used to define a negligible imbalance is a SMD <10% [11, 12]. However, even in case of a variable with a SMD>10% after the matching procedure, a potential imbalance by the SMD still needs to be assessed in its clinical context and relevance.

Only few variables did not yield a SMD<10% after the matching procedure. Sex, BMI, ARDS etiology, and treatment with inhaled nitric oxide were of overall low prognostic relevance and were a priori not used for the matching procedure. Prone positioning, extracorporeal life support,

and ECMO sweep gas flow had a SMD slightly higher than 10% but as prognostic determinants they were included in the matching procedure. However, these variables showed no relevant differences when the two groups were compared. All other prognostic determinants that were included in the matching procedure had a very low SMD <10%. The jitter plot of the matching diagnostics (Figure S3) visualizes that the prognostic determinants were well balanced between the two hemoglobin threshold groups.

Data sources

Data on patients' demographics and comorbidities were extracted from the hospital data management system (SAP, Walldorf, Germany). Data on admission scores, ARDS characteristics, ARDS treatment, rescue therapies, supportive therapies, medications, ventilation parameters, ECMO, transfusion, and laboratory parameters such as hemoglobin measurements were extracted from the electronic intensive care unit data management system in use at the hospital (COPRA 5, Sasbachwalden, Germany).

Supplemental Tables

Table S1: Baseline characteristics of the unmatched cohort.

Characteristic	Higher-threshold group (N=128)	Lower-threshold group (N=240)	P-value	SMD
**Age** (years)	51.5 (37.0 - 63.2)	53.0 (41.0 - 64.0)	0.11	0.195
**Male sex**, n (%)	83 (64.8)	154 (64.2)	0.91	0.014
**Body mass index** (kg/cm)	26.3 (23.6 - 31.2)	26.2 (23.1 - 30.4)	0.26	0.157
**Charlson comorbidity index**	2.0 (1.0 - 4.0)	3.0 (1.0 - 5.0)	0.08	0.220
**Immunocompromised**, n (%)	26 (20.3)	61 (25.4)	0.30	
**Year of admission**, n (%)			<0.001	2.024
2007-2010	57 (44.5)	15 (6.2)		
2011-2014	61 (47.7)	39 (16.2)		
2015-2018	10 (7.8)	186 (77.5)		
**SOFA at ARDS onset**	12.0 (9.0 - 16.0)	11.0 (9.0 - 14.0)	0.08	0.195
**SAPS II at ARDS onset**	58.5 (40.0 - 71.0)	57.0 (42.0 - 68.0)	0.89	0.037
**RASS at ARDS onset**	-5.0 (-5.0 - -4.0)	-5.0 (-5.0 - -4.0)	0.70	0.157
**Chronic lung disease**, n (%)	38 (29.7)	65 (27.1)	0.63	0.058
**Pulmonal origin**, n (%)	104 (81.2)	199 (82.9)	0.77	0.043
**Mechanical ventilation before admission** (days)	2.0 (1.0 - 7.0)	1.0 (1.0 - 5.0)	0.30	0.127
**ARDS severity**, n (%)			0.003	0.396
Mild	0 (0.0)	8 (3.3)		
Moderate	11 (8.6)	43 (17.9)		
Severe	117 (91.4)	189 (78.8)		
**ARDS etiology**, n (%)			0.86	0.132
Pneumonia	81 (63.3)	145 (60.4)		
Aspiration	15 (11.7)	37 (15.4)		
Sepsis	9 (7.0)	14 (5.8)		
Pancreatitis	3 (2.3)	8 (3.3)		
Other	20 (15.6)	36 (15.0)		
**Rescue therapy**				
Inhaled Nitric oxide, n (%)	112 (87.5)	142 (59.2)	<0.001	0.676
Prone positioning, (%)	99 (77.3)	162 (67.5)	0.05	0.222
Extracorporeal life support, n (%)			<0.001	0.603
No ECLS	38 (29.7)	113 (47.1)		
ECLA	18 (14.1)	8 (3.3)		
ECMO	59 (46.1)	115 (47.9)		
Combined	13 (10.2)	4 (1.7)		
**Ventilation parameters after initial optimization**				
PaO2:FiO2 (mmHg)	125 (91 - 163)	168 (118 - 233)	<0.001	0.547
Oxygenation index	18.2 (13.1 - 27.5)	12.8 (8.6 - 20.7)	<0.001	0.636
PEEP (cm H2O)	16.3 (14.4 - 19.5)	16.3 (12.3 - 18.5)	0.06	0.309
Driving pressure (cm H2O)	15.9 (13.0 - 19.6)	14.9 (11.7 - 17.7)	0.011	0.322
Tidal volume (ml/kg PBW)	5.5 (3.9 - 7.2)	5.9 (4.3 - 7.1)	0.64	0.022
Compliance (ml/cm H2O)	26 (17.7 - 37.5)	32 (22.1 - 43.1)	0.004	0.312
**ECMO initiation (ICU day)**	0 (0 - 0)	0 (0 - 0)	0.25	0.110
**ECMO pump flow** (l/min)	3.8 (3.2 - 4.4)	3.5 (2.8 - 4.0)	0.007	0.455
**ECMO ventilation** (l/min)	5.0 (3.9 - 7.0)	3.5 (2.5 - 5.0)	<0.001	0.434
**Septic shock**, n (%)	75 (58.6)	113 (47.9)	0.06	0.216
**Lactate** (mg/dl)	20.0 (13.8 - 53.2)	17.0 (11.0 - 38.0)	0.02	0.252
**pH**	7.3 (7.2 - 7.4)	7.3 (7.2 - 7.4)	0.17	0.184
**RRT**, n (%)	155 (64.6)	83 (64.8)	0.76	0.005

Definition of abbreviations: SOFA = Sequential Organ Failure Assessment, SAPS = Simplified Acute Physiology Score, RASS = Richmond Agitation-Sedation Scale, ECLS = Extracorporeal life support, ECLA = pumpless extra corporal lung assist, ECMO = Extracorporeal membrane oxygenation, PEEP = Positive End-Expiratory Pressure, PBW = Predicted body weight, ICU = Intensive care unit, RRT = Renal replacement therapy.

Data are expressed as median [25%, 75% quartiles] or frequencies [%], as appropriate. P-values were calculated using the exact Wilcoxon-Mann-Whitney test and the Fisher's exact test, as appropriate. Standardized mean differences (SMD) are provided.

Supplemental Figures

Figure S1: Transfusion practice and mortality during the study period. Individual hemoglobin threshold for RBC transfusion (A) and 28-day mortality (B) during the study period (year of admission 2007 to 2018). The hazard ratio (with 95%-CI) for the year of admission as continuous variable is indicated.

A. Individual hemoglobin threshold for RBC transfusion

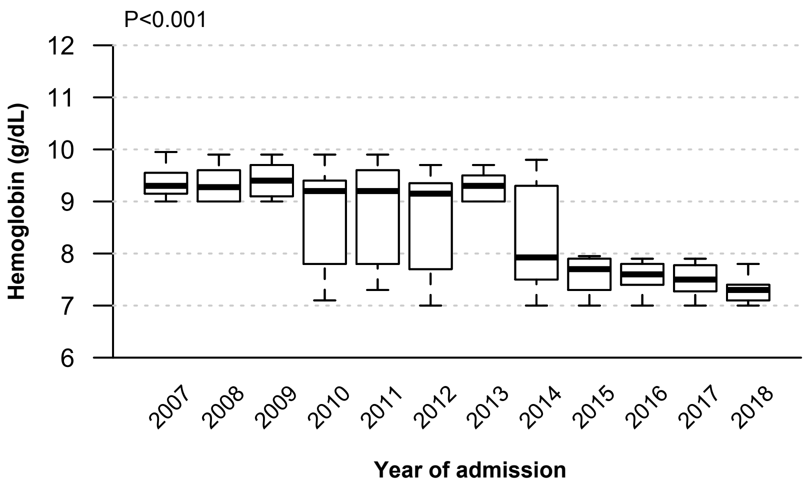

B. Mortality

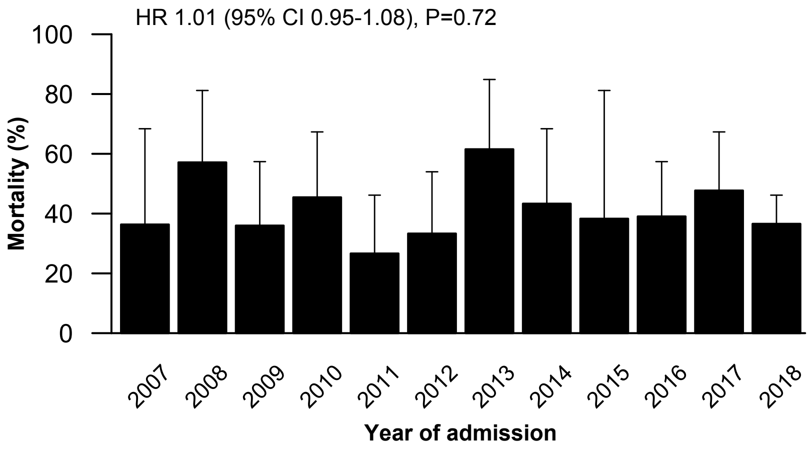

Figure S2: Distribution of propensity scores. Propensity scores of the lower-threshold and higher-threshold group are presented for all patients (A), the matched cohort (B), and the cohort of patients who could not be matched due to no matching partner available (C). The mean PS (with SD) in each cohort is indicated.

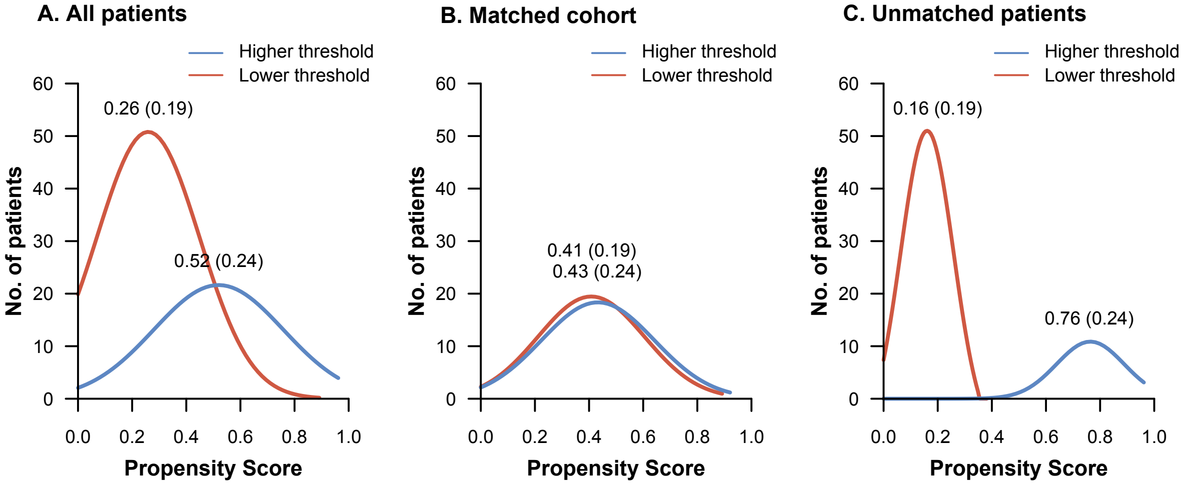

Figure S3: Distribution of propensity scores – detailed visualization. Propensity scores of the lower-threshold and higher-threshold group are presented using a jitter plot for the unmatched and matched patients. Each circle represents a patient.

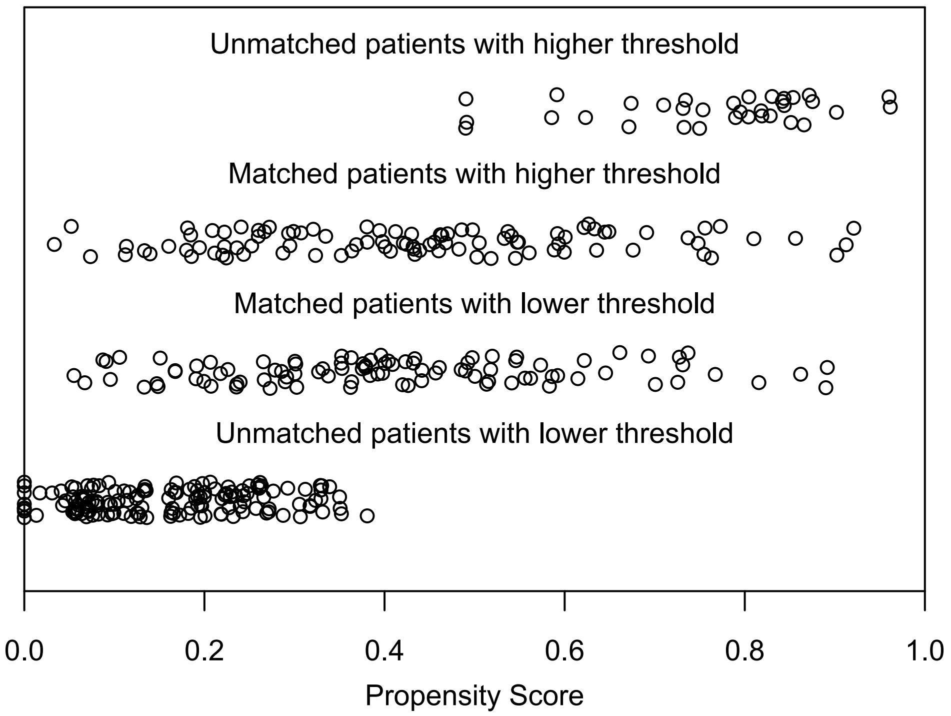

Figure S4: Daily transfusion requirements. Number of RBC units transfused during the 28-day period after ARDS onset between the lower-threshold and higher-threshold group.

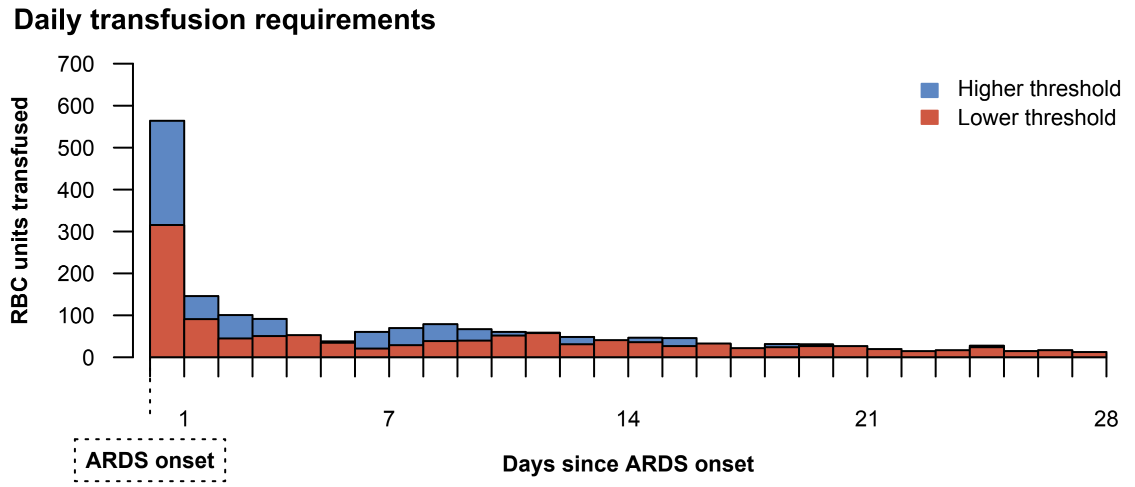

Figure S5: Additional failure-free composites. Cumulative incidence curves of renal replacement-free (RRT) (A), and vasopressor-free days (B) composites between the lower-threshold and higher-threshold group.

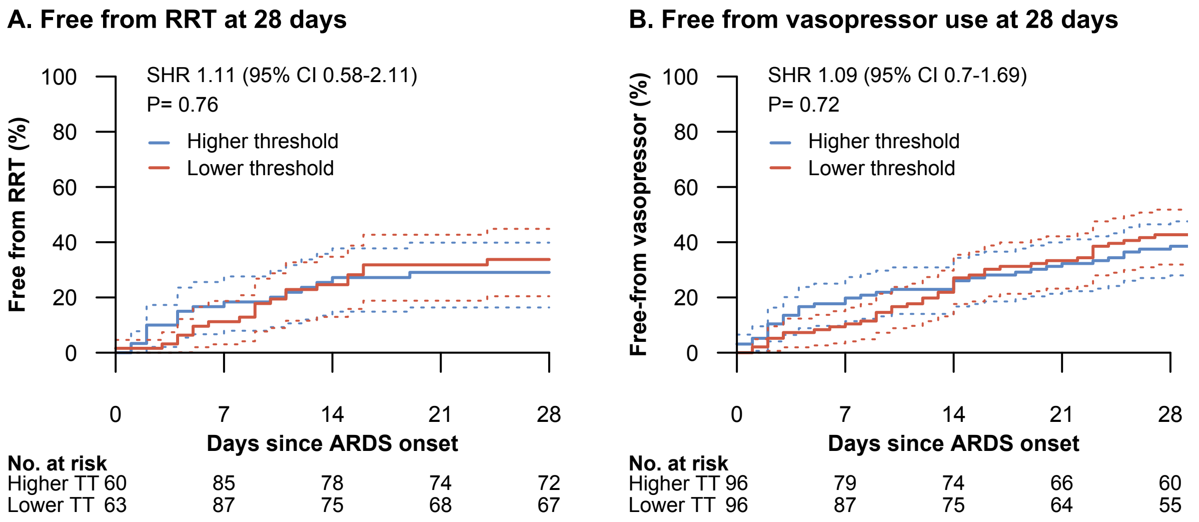

Figure S6: Determinants of gas exchange and acid-base status. Median daily time-weighted values are presented during 28 days of ARDS therapy for each threshold group.

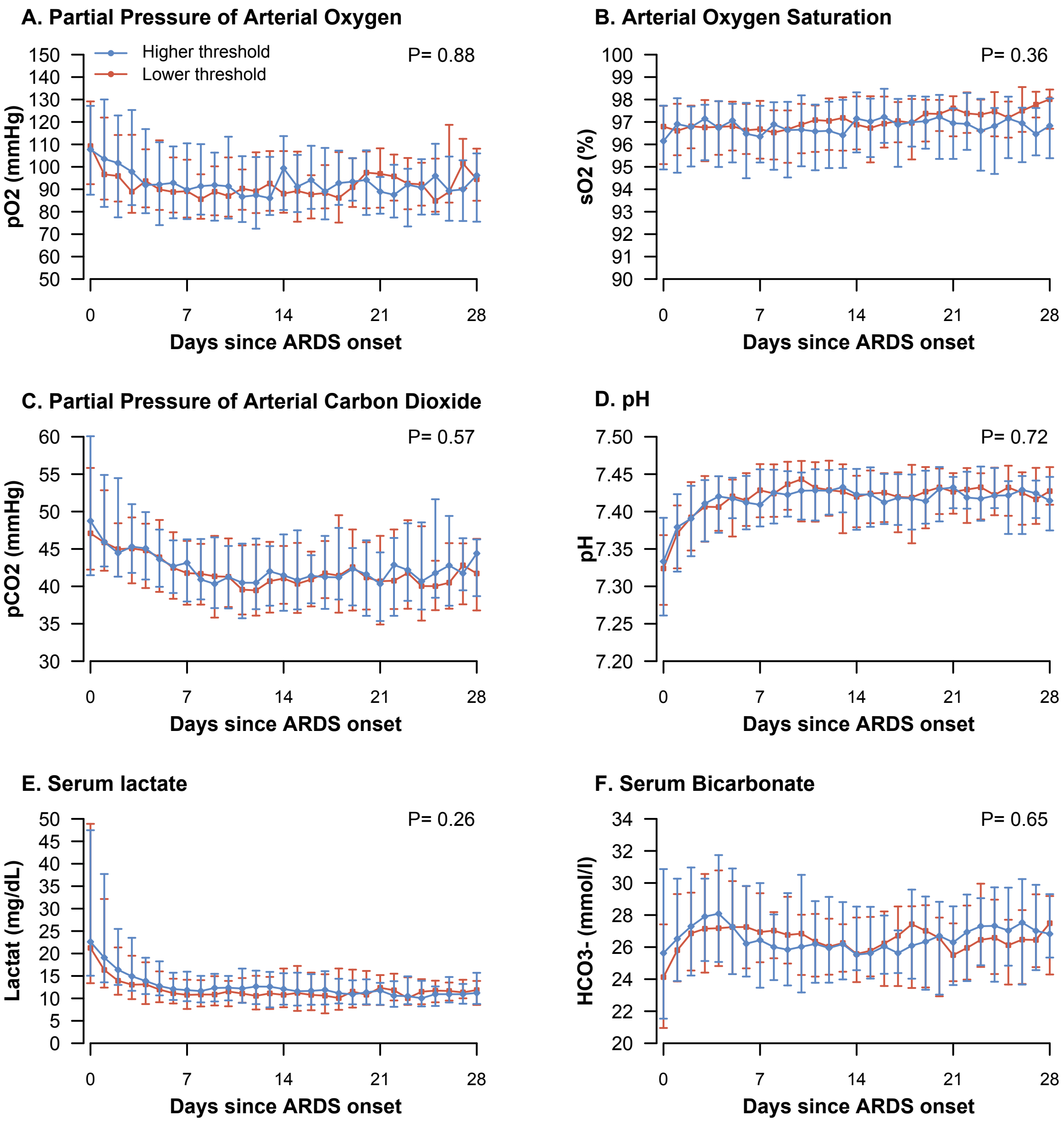

Daily time-weighted values overcome the complexity that number and timing of daily blood gas samples were not exactly the same in all patients. First values were the baseline hemoglobin concentrations at ARDS onset. Day 0 was defined as the time of ARDS onset to the end of that day. Data are shown as median and 25th and 75th percentiles.

References

1. Finney SJ, Zekveld C, Elia A, Evans TW: **Glucose control and mortality in critically ill patients**. *JAMA* 2003, **290**(15):2041-2047.
2. Carson JL, Guyatt G, Heddle NM, Grossman BJ, Cohn CS, Fung MK, Gernsheimer T, Holcomb JB, Kaplan LJ, Katz LM *et al*: **Clinical Practice Guidelines From the AABB: Red Blood Cell Transfusion Thresholds and Storage**. *JAMA* 2016, **316**(19):2025-2035.
3. Carson JL, Stanworth SJ, Roubinian N, Fergusson DA, Triulzi D, Doree C, Hebert PC: **Transfusion thresholds and other strategies for guiding allogeneic red blood cell transfusion**. *Cochrane Database Syst Rev* 2016, **10**:CD002042.
4. Mueller MM, Van Remoortel H, Meybohm P, Aranko K, Aubron C, Burger R, Carson JL, Cichutek K, De Buck E, Devine D *et al*: **Patient Blood Management: Recommendations From the 2018 Frankfurt Consensus Conference**. *JAMA* 2019, **321**(10):983-997.
5. Vlaar AP, Oczkowski S, de Bruin S, Wijnberge M, Antonelli M, Aubron C, Aries P, Duranteau J, Juffermans NP, Meier J *et al*: **Transfusion strategies in non-bleeding critically ill adults: a clinical practice guideline from the European Society of Intensive Care Medicine**. *Intensive Care Med* 2020, **46**(4):673-696.
6. Yehya N, Harhay MO, Curley MAQ, Schoenfeld DA, Reeder RW: **Re-appraisal of Ventilator-free Days in Critical Care Research**. *Am J Respir Crit Care Med* 2019.
7. Devlin JW, Skrobik Y, Gelinas C, Needham DM, Slooter AJC, Pandharipande PP, Watson PL, Weinhouse GL, Nunnally ME, Rochwerg B *et al*: **Clinical Practice Guidelines for the Prevention and Management of Pain, Agitation/Sedation, Delirium, Immobility, and Sleep Disruption in Adult Patients in the ICU**. *Crit Care Med* 2018, **46**(9):e825-e873.
8. Muller A, Weiss B, Spies CD, Leitliniengruppe S: **["Symptomatic Treatment of Delirium, Anxiety and Stress, and Protocol Based Analgesia, Sedation and Management of Sleep in Intensive Care Patients"]**. *Anasthesiol Intensivmed Notfallmed Schmerzther* 2015, **50**(11-12):698-703.
9. Annane D, Renault A, Brun-Buisson C, Megarbane B, Quenot JP, Siami S, Cariou A, Forceville X, Schwebel C, Martin C *et al*: **Hydrocortisone plus Fludrocortisone for Adults with Septic Shock**. *N Engl J Med* 2018, **378**(9):809-818.
10. Azoulay E, Lemiale V, Mourvillier B, Garrouste-Orgeas M, Schwebel C, Ruckly S, Argaud L, Cohen Y, Souweine B, Papazian L *et al*: **Management and outcomes of acute respiratory distress syndrome patients with and without comorbid conditions**. *Intensive Care Med* 2018, **44**(7):1050-1060.
11. Austin PC: **A comparison of 12 algorithms for matching on the propensity score**. *Stat Med* 2014, **33**(6):1057-1069.
12. Normand ST, Landrum MB, Guadagnoli E, Ayanian JZ, Ryan TJ, Cleary PD, McNeil BJ: **Validating recommendations for coronary angiography following acute myocardial infarction in the elderly: a matched analysis using propensity scores**. *J Clin Epidemiol* 2001, **54**(4):387-398.
